# Fabrication of chemically stable hydrogen- and niobium-codoped ZnO transparent conductive films†

**DOI:** 10.1039/c9ra01231a

**Published:** 2019-04-24

**Authors:** Bing Han, Jianmin Song, Junjie Li, Yajuan Guo, Binting Dai, Xudong Meng, Weiye Song, Fu Yang, Yanfeng Wang

**Affiliations:** Institute of New Energy Science and Technology of Hebei North University Zhangjiakou 075000 China yanfengwangnk@163.com; College of Sciences, Agriculture University of Hebei Baoding 071001 China

## Abstract

H- and Nb-doped ZnO (HNZO) thin films were fabricated on glass substrates with radio frequency magnetron sputtering. The effect of the flow rate of H_2_ has been investigated by analyzing the structural, optical, and electrical properties. The incorporation of H during the deposition of Nb-incorporated ZnO films significantly improved their crystallinity, conductivity, and transmittance. The crystallites of the HNZO films were preferentially oriented in the *c*-axis direction; the films possess high transmittance (approximately 85%) in the visible and near-infrared regions (400 to 1400 nm). The lowest room-temperature resistivity of the HNZO films was measured as 1.28 × 10^−3^ Ω cm. Such optical and electrical properties along with the remarkable chemical stability of the HNZO films make them a promising candidate for applications in solar cells.

## Abstract 2

H- and Nb-doped ZnO (HNZO) thin films were fabricated on glass substrates with radio frequency magnetron sputtering.

## Graphical abstract

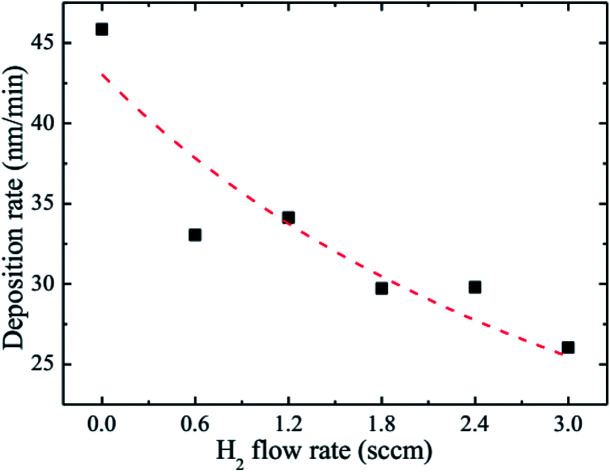

## 1. Introduction

Thanks to their inherent characteristics such as high electrical conductivity and high optical transmittance in the visible region, transparent conducting oxide (TCO) films have found many applications as transparent electrodes in solar cells, optoelectronic devices, flat-panel displays, and light-emitting diodes.^1,2^ Any further extensive application of indium tin oxide (ITO) films, such as the most commonly used TCO material, is hindered by the fact that indium is rare, expensive, and toxic. Therefore, impurity-doped ZnO films have been considered as alternative TCO films, which is because of their superior electrical and optical properties complementing their low cost and non-toxicity.^3–5^ Moreover, exhibiting high electrical and optical stability under hydrogen plasma, ZnO thin films have been used as front electrodes in silicon thin-film solar cells.^6–8^

Al is the most widely studied dopant for ZnO films. Fabricated Al-doped ZnO films exhibit electric resistivity as low as 2–3 × 10^−4^ Ω cm and optical transmittance higher than 80% in the visible range.^9–11^ However, free-carrier absorption in the near-infrared (NIR) region is the main drawback of Al-doped ZnO films, which limits their long-wavelength transmittance, which consequently limits the long-wavelength response of μc-Si:H-based Si thin-film solar cells.^9–11^

It has previously been shown that TCO films doped with an ion that has a valence largely different than that of the film material possess both low resistivity and wide-spectrum transmittance, which is because of less-pronounced ion scattering effect as compared to other doped oxides at the same carrier concentration.^12,13^ The ionic radius of Nb^5+^ (0.070 nm) is smaller than that of Zn^2+^ (0.074 nm), and their valence difference is large. Furthermore, the high thermal stability of Nb is even further enhanced when doped into ZnO.^14^ Therefore, Nb is an ideal donor in ZnO films-it can provide enough free carriers to improve the film's conductivity at very small concentrations.^15^

Co-doping has been widely employed to further improve the electrical and optical properties of ZnO films. For example, band-gap widening and good conductivity of ZnO films were achieved by their co-doping with group-III elements (Al, Ga, or In) and Mg.^16–20^ Compared with Al-doped ZnO films (AZO), the ZnO films which were codoped with Al and another element from group III (B, Ga, or In) exhibit lower resistivities.^21–23^ Though less-considered, the cation–anion co-doping of ZnO films is worth exploring; the ZnO thin films codoped with N and a group-III element (B, Al, or Ga) have a p-type conductivity.^24–26^ As found by recent first-principle calculations, for any Fermi-level position, H^+^ in ZnO has a lower energy than H^0^ and H^−^, which suggests that hydrogen in ZnO can act as shallow n-type donor-a unique doping characteristic which is different from the amphoteric function that hydrogen has in semiconducting or insulating materials.^27–29^ Triggered by this finding, many experimental studies confirmed that the hydrogen doping of pure ZnO and metal-doped ZnO films can improve their electrical properties and stability in air.^30–41^

Therefore, it is expected that codoping allows ZnO films to benefit from combined effects of H and Nb dopants, which has not been experimentally demonstrated yet. Here we report on the fabrication of H-and-Nb-codoped ZnO (HNZO) films in an atmosphere of Ar and H_2_ with different hydrogen flow rates which allow for investigating the effect of hydrogen flux on structural, electrical, and optical properties of Nb-doped ZnO (NZO) films. The chemical stability of the fabricated HNZO in diluted HCl and NaOH was also investigated.

## 2. Experimental

The HNZO films were deposited on glass substrates (Corning XG) using the radio-frequency magnetron sputtering (RFMS) technique under various atmospheres of Ar and H_2_ at room temperature (RT). During deposition, the substrate was kept at constant temperature. The target—a ceramic disc of ZnO (99.99% pure) mixed with 2 wt% of Nb_2_O_5_ (99.99% pure)-was fixed at the distance of 5 cm away from the substrate which was ultrasonically cleaned with a detergent, rinsed with deionized water, and subsequently dried with nitrogen gas. The background pressure of the reaction chamber was kept at less than 5 × 10^−5^ Pa. Ar and H_2_ gases were fed into the chamber through mass flow controllers, maintaining the working pressure at 0.13 Pa. Different flow rates of H_2_ (0, 0.6, 1.2, 1.8, 2.4, and 3.0 sccm) and Ar (30.0, 29.4, 28.8, 28.2, 27.6, and 27 sccm) were used to examine the effect of H_2_ on the properties of the NZO films. The working power of the radio-frequency magnetron was fixed at 180 W; the film thickness was approximately 1000 nm.

An commercially available AZO (1000 nm) films with sheet resistance of 7.8 Ω □^−1^ were used as reference to compare the chemical durability with that of an optimal HNZO film that exhibited the best electrical and optical properties, both of these films were etched at room temperature with diluted HCl (0.5%), and at 80 °C with NaOH (5%) for different etching times. X-Ray diffraction (XRD, Rigaku, ATX-XRD) was used to determine the crystal structure of the HNZO films. The transmittance spectra were recorded within the range of 300–1400 nm using a spectrophotometer (Shimadzu UV-3600 Plus). Van der Pauw method (HMS-3000 Hall System) at room temperature was used to measure the electrical properties. The chemical state of the surface of the HNZO films was analyzed with high-resolution X-ray photoelectron spectrometry (XPS, Kratos Axis Ultra DLD multi-technique). All normal XPS spectra were calibrated by the C 1s peak (∼285 eV) from contamination.

## 3. Results and discussion

**Fig. 1:**
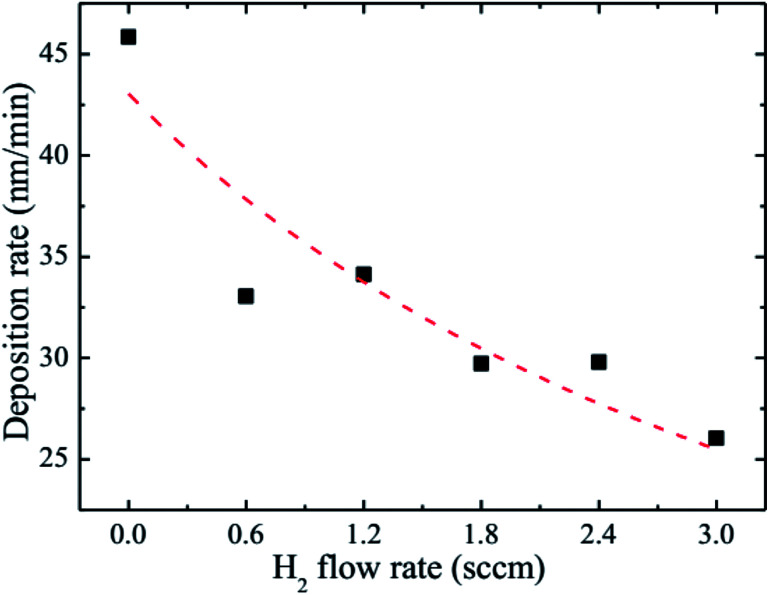
The deposition rate of the HNZO films as a function of the flow rate of H_2_.

The observed decrease of the deposition rate of the HNZO films with the increase in the flow rate (FR) of H_2_ (Fig. 1) can be attributed to the gas dilution effect, and to possible hydrogen–oxygen chemical reactions within the plasma ambient.^42^

**Fig. 2:**
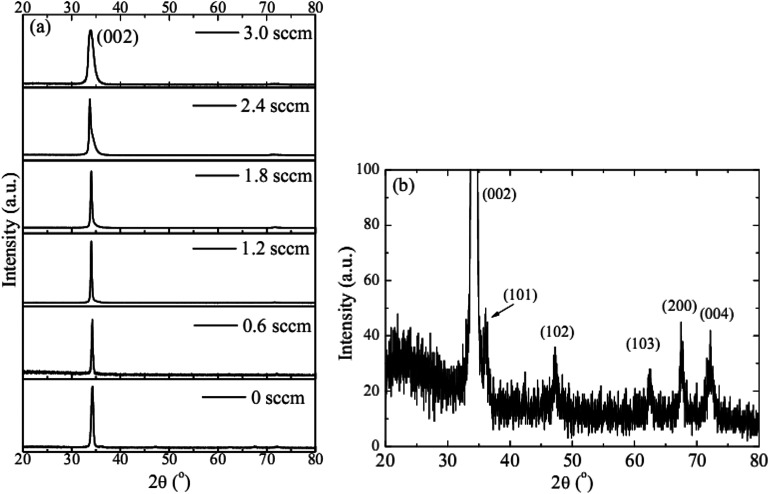
(a) The XRD patterns of the HNZO films as a function of the flow rate of H_2_, (b) the amplified XRD pattern of the NZO film (0 sccm of H_2_).

**Fig. 3:**
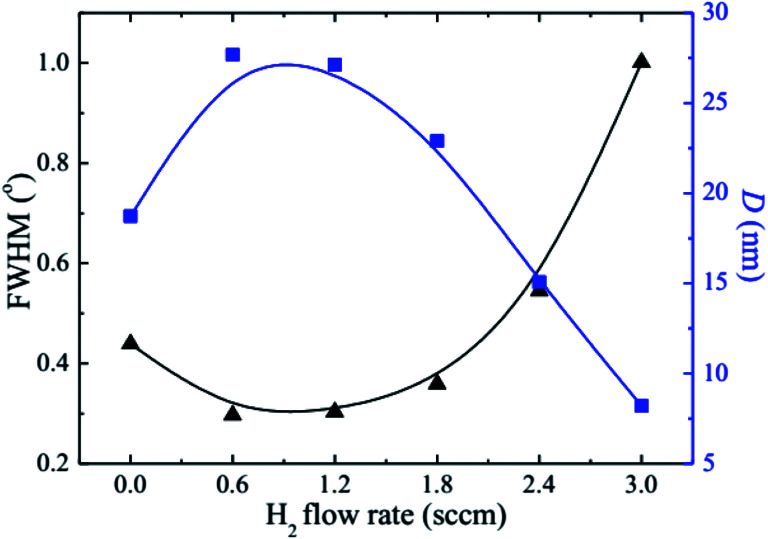
The FWHM and crystallite size (*D*) of the HNZO films as a function of the flow rate of H_2_.

As inferred from the XRD patterns given in Fig. 2 as a function of the flow rate of H_2_, all the HNZO films exhibited a (002) diffraction peak around 34°—which is an indication of a typical wurtzite structure whose *c*-axis was preferentially perpendicular to the substrate. The XRD patterns does not show any indication of the presence of any phase which corresponds to the dopants, which is either a reflection of the substitution of the incorporated Nb ions for Zn ions in the hexagonal lattice or an indication of the segregation of the Nb ions into non-crystalline regions at the grain boundaries. The XRD pattern of the NZO film (0 sccm of H_2_) shows other peaks such as (101), (102), (103) and (200) besides the dominant (002) and (004) peaks (Fig. 2(b)). These structural observations are consistent with self-textured ZnO films prepared by low-pressure chemical vapor deposition method^43^ or ultrasonic spray pyrolysis method.^44^ The other abovementioned diffraction peaks can be attributed to the creation of a pyramid-like surface morphology (see Fig. 2(b)). The intensity of the (002) reflection was found to initially decrease upon the introduction of H_2_, and to subsequently increase when the flow rate of H_2_ was increased to 1.2 sccm; however, for H_2_ flow rates beyond this value, the (002) orientation was less favored. This observation can be explained by taking the high-energy O^2−^ ion bombardment of the NZO film into account; that is, the ion bombardment damages the (002) planes more seriously than other loosely-packed planes such as (101)—the surface of the growing film will have a large number of dangling bonds;^45,46^ therefore, the growth of the crystallites that are normal to the (101) plane will continue relatively undisturbed, and they can serve as seeds for further growth;^45,46^ however, the termination of the dangling bonds upon the incorporation of hydrogen enables the sputtered atoms to travel further. Upon the hydrogen doping, the microstructure of the NZO film changes from an unstable to a more stable orientation, *i.e.* (101) or (200) to (002) plane growth—thanks to the lowest surface energy of the (002) crystal plane.^47^ H_2_ flow rates more than 1.2 sccm can considerably reduce the (002) peak intensity; that is, the H dopant concentration in excess of the saturation stage deteriorates the crystal quality of the NZO films, and arrests the (101) and (200) plane growth.^48^Fig. 3 presents the full-width-at-half-maximum (FWHM) of the (002) peak, and the grain size (*D*) calculated according to Scherrer formula^49^1
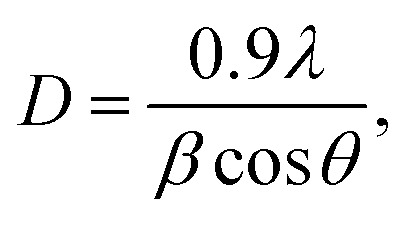
where *λ* is the X-ray wavelength, *β* is the FWHM of the considered peak, and *θ* is the Bragg angle. When the H dopant concentration increases, the FWHM decreases initially to a minimum value of 0.30° at the H_2_ flow rate of 0.6 sccm, and subsequently increases to a maximum value of 1.0° when the H_2_ flow rate raises to 3.0 sccm. The trend of the flow-rate-dependence of *D* is opposite to that of the FWHM; that is, an increase in the FWHM corresponds to decrease in the grain size; therefore, the H_2_ flow rate should be controlled within the small range of 0.6–1.2 sccm in order for the NZO thin films to grow with good crystallinity.

**Fig. 4:**
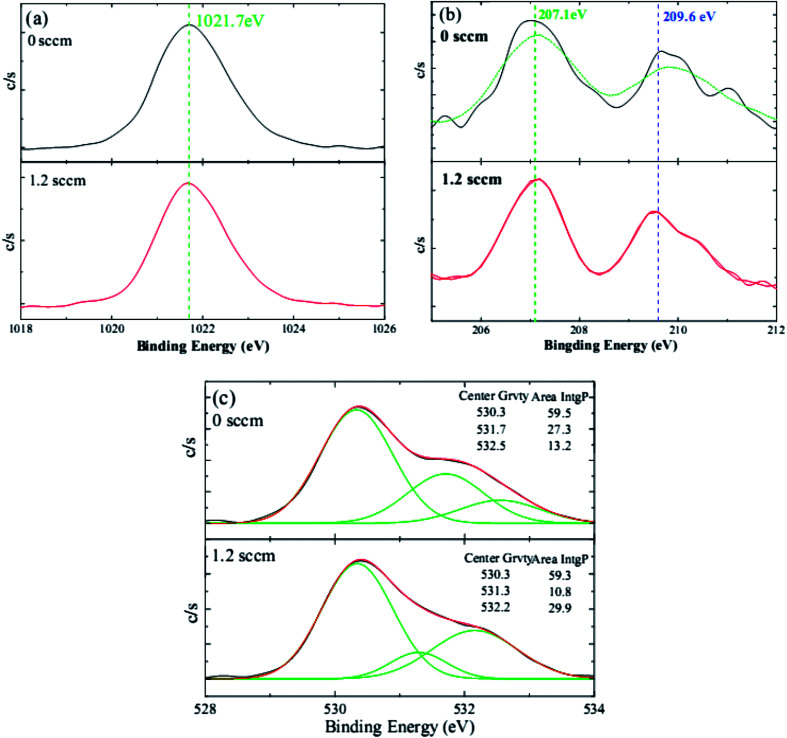
The typical XPS spectra of (a) Zn 2p, (b) Nb 3d, and (c) O 1s of the NZO films with and without H doping.

To better understand the doping mechanism of H into the NZO films, the valence states Zn, Nb, and O of one NZO film and one HNZO film that was fabricated at the hydrogen flow rate of 1.2 sccm were inspected using XPS measurements. Fig. 4 shows the typical XPS spectra of Zn, Nb, and O. The symmetry of the core lines of Zn 2p3/2 located at 1021.7 ± 0.1 eV (ref. 50 and 51) suggest that Zn atoms mainly remain in the same formal valence state of Zn^2+^ in both the NZO and HNZO films—the incorporated H does not influence the combined state of Zn. Consistent with the aforementioned XRD analysis, the XPS spectrum does not show any indication of the presence of metallic Zn peak, which reflects that the Zn exists only in the oxidized state. Moreover, the two peaks of Nb 3d5/2 and 3d3/2 at respectively 207.1 ± 0.1 eV (ref. 52) and 209.6 ± 0.2 eV (ref. 53) indicate that the Nb is in its oxidation state (Nb^5+^), takes the place of the Zn^2+^ in the NZO film in the form of an Nb–O bond, and becomes a substitute for the Zn^2+^ in the HNZO film as an Nb–O or Nb–H–O bond.^54^ Three O peaks are observed in the XPS spectrum for both the NZO and HNZO films (Fig. 4(c)). The low-energy component of the NZO film at 530.4 ± 0.1 eV is attributed to the oxygen ions surrounded by the Zn (or by substitutional Nb) atoms,^55^ whereas the peak at the higher binding energy of 531.7 ± 0.4 eV is due to the oxygen ions within those regions of the ZnO matrix that are deficient of oxygen.^56,57^ The peak with the highest binding energy that is located at 532.4 ± 0.2 eV corresponds to chemisorbed or dissociated oxygen or hydroxyl species such as adsorbed O_2_.^58^Fig. 4(c) shows that the incorporation of hydrogen makes the integral area of the middle-binding-energy peak decrease from 27.3% to 10.8%, and the integral area of the peak with the highest binding energy increase from 13.2% to 29.9%. Such change is put down to the formation of O–H bonding as Van de Walle predicted by first-principle calculations.^27,58^

**Fig. 5:**
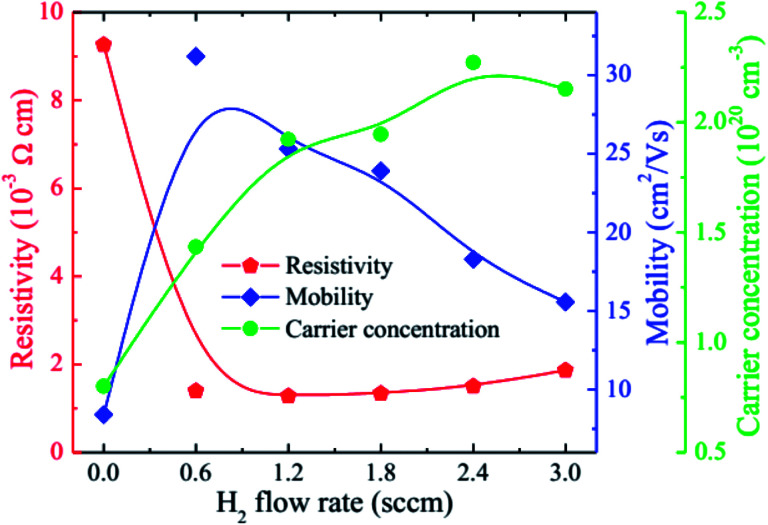
The carrier concentration (*n*), mobility (*μ*), and resistivity (*ρ*) of the HNZO films as functions of the flow rate of H_2_.

Fig. 5 gives the carrier concentration (*n*), mobility (*μ*), and resistivity (*ρ*) of the HNZO films as a function of the flow rate of H_2_. The resistivity of the unhydrogenated NZO film (9.26 × 10^−3^ Ω cm) is lower than that of the pure ZnO film, which is mainly attributed to the substitutional effect of Nb^5+^ ions on the remaining Zn^2+^ ions (Fig. 4(b)). Upon the incorporation of H, the resistivity of the NZO film is markedly lowered than that of the pure NZO film. When the hydrogen flow rate was increased, the resistivity was first decreased, and was then maximized when the hydrogen flow rate reached 1.2 sccm; this is attributed to the increase in both *n* and *μ*, thanks to a small amount of hydrogen doping. Further increase of the flow rate of H_2_ to 2.4 sccm led to *n* reaching a maximum, and to *μ* decreasing monotonically. The formation energy of the O–H stretch (in the H^+^ form) in the Zn–O is the lowest, and the doped hydrogen in ZnO films is an n-type donor.^27,59^ Furthermore, hydrogen can be interstitially incorporated into ZnO-related films, and can form dangling bonds in grain boundaries.^60^ The increase in *n* upon the incorporation of hydrogen is attributed to the formation of the O–H stretch in Zn–O bonds. The decrease in *μ* with increasing the flow rate of H_2_ may be put down to ionized impurity scattering and grain boundary scattering that could be because of more hydrogen atoms and smaller grain size in the films (Fig. 3). We found that the optimal value of the H_2_ flow rate is 1.2 sccm with *ρ* being 1.28 × 10^−3^ Ω cm, *n* being 1.92 × 10^20^ cm^−3^, and *μ* being 25.32 cm^2^ V^−1^ s^−1^. The beneficial effect of hydrogen doping was also observed in fluorine-doped ZnO films.^42^

**Fig. 6:**
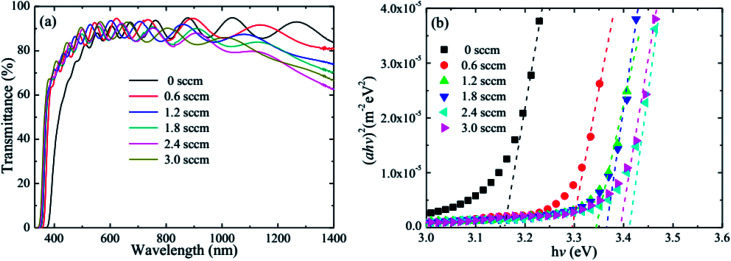
(a) The optical transmittance, and (b) optical band gap of the HNZO films as a function of the flow rate of H_2_.

Fig. 6 shows the optical transmittance and optical band gap of the HNZO films as a function of the H_2_ flow rate. The optical transmittance strongly depends on the flow rate. When flow rate increases, the absorption edge of the film shifts to the short wavelength region, being an indication of the broadening of the optical band gap of the HNZO films; the transmittance is also significantly improved around 400 nm. This is understood by the Burstein–Moss effect—the upward shift of the Fermi level inside the conduction band because of an increased donor concentration which leads to the conduction band being further filled by an increased number of electron carriers.^61^ Although the hydrogen-induced increase of carrier concentration reduces the long-wavelength transmittance, the transmittance of the HNZO films in the range of 400–1400 nm is on average about 85%.

The optical band gap (*E*_g_) as a function of the flow rate is determined by applying the Tauc model in the high-absorbance region^62^

2 *αhv* = *D*(*hv* − *E*_g_)^*n*^,

where *hv* is the photon energy, and *D* is a constant. For *n* = 1/2, the transition data provided the best linear curve in the band-edge region, which indicates that the transition was direct in nature. The band gaps of the films were then calculated using the Tauc model by plotting (*αhv*)^2^ with respect to *hv*, and extrapolating the linear portion of the absorption edge to find the intercept with the energy axis. Fig. 6(b) gives the *E*_g_ of the films as a function of the flow rate. The band gap was first maximized at 3.41 eV for the flow rate of 2.4 sccm, and was then lowered when the flow rate was further increased. In other words, the variation of the optical properties of the HNZO films is due to the change of *n* (Fig. 5) made by the H doping. Considering the electrical and optical properties of the HNZO films, we determine the optimal value of the H_2_ flow rate as 1.2 sccm with the lowest *ρ* of 1.28 × 10^−3^ Ω cm, and the average transmittance of about 85% in the range of 400–1400 nm, which is satisfactory on the basis of the rigorous application requirements of high-efficiency thin-film solar cells.^63^

In order that the special requirements of thin-film solar cells for front contact are met, the TCO films should also enjoy high chemical stability besides they exhibit good optical and electrical properties. We tested the chemical stability of the abovementioned electrically- and optically-optimal HNZO films by etching them in diluted HCl and NaOH for different time periods. For comparison, the sputtered AZO films were also etched using the same experimental procedures.

**Fig. 7:**
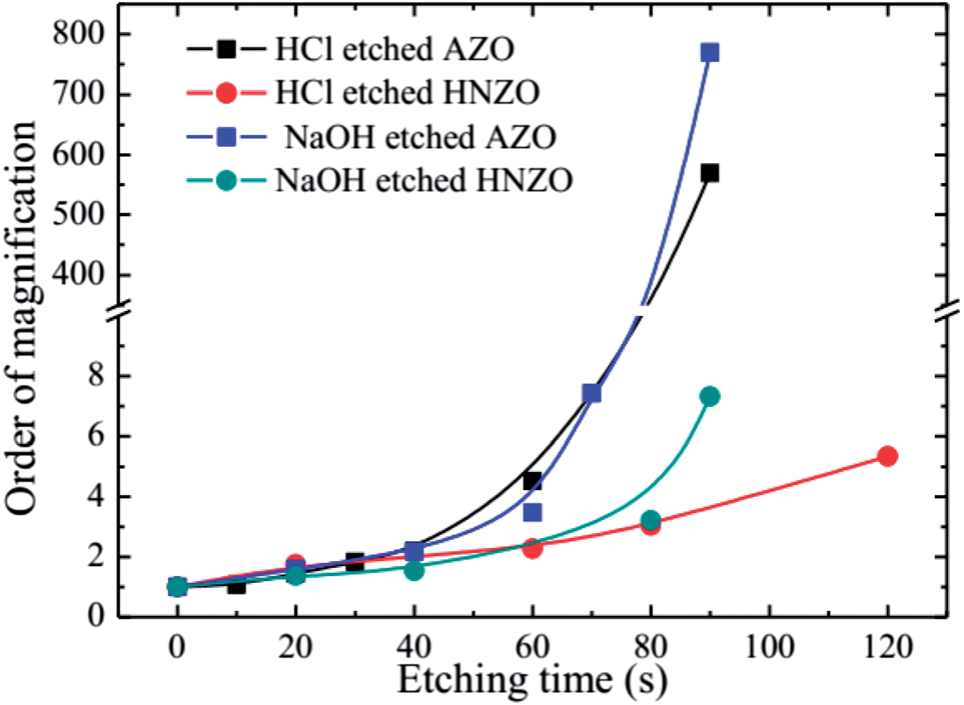
The multiple changes of the sheet resistance of the HCl- and NaOH-etched AZO and HNZO films at different etching times.

Fig. 7 shows multiple changes of sheet resistance for the etched AZO and HNZO films. The sheet resistances of the as-deposited AZO and HNZO films were 7.8 Ω □^−1^ and 13.5 Ω □^−1^, respectively. As shown in Fig. 7, almost no changes were observed in both the AZO and HNZO samples etched for less than 40 seconds in either HCl or NaOH solutions. However, when the samples were etched for more than 40 seconds, the order of magnification was sharply increased for both the HCl- and NaOH-etched AZO films. On the contrary, the corresponding changes for both of the HCl- and NaOH-etched HNZO films are relatively small. When the etching time of the HCl-etched HNZO film was increased from 60 s to 120 s, its order of magnification was increased only from 2.3 to 5.3; this change of order of magnification for the NaOH-etched HNZO film was from 3.2 to 7.3 when the etching time was increased from 80 s to 90 s. Therefore, the HNZO films are highly chemically-stable, which demonstrates their potential for broad applications in thin-film solar cells.

## 4. Conclusion

In this study, transparent conducting HNZO films were deposited using the RFMS method on glass substrates. The structural, electrical, and optical properties of the NZO films were investigated as a function of the H_2_ flow rate. All of the as-deposited films possessed good crystallinity with a *c*-axis preferential orientation, low room-temperature resistivity, and high broadband spectrum transmittance of more than 85% at the range of 400 to 1400 nm. The lowest resistivity of 1.28 × 10^−3^ Ω cm, with a Hall mobility of 25.32 cm^2^ V^−1^ s^−1^, and an electron concentration of 1.92 × 10^20^ cm^−3^ were achieved at the H_2_ flow rate of 1.2 sccm. Furthermore, the carrier concentration was mainly originated from the substitution of Nb^5+^ for Zn^2+^, and from the formation of Zn–H–O and Nb–H–O bonds. The HNZO films have high acid and alkali resistance, which demonstrates their potential for broad applications in optoelectronic devices.

## Conflicts of interest

There are no conflicts to declare.

## Supplementary Material

RA-009-C9RA01231A-s001
