# Supplementary material for: Fabrication of chemically stable hydrogen- and niobium-codoped ZnO transparent conductive films
Source: RSC Adv. 2019 Apr 24;9(22):12681–8. doi: 10.1039/c9ra01231a (PMC9063663; doi:10.1039/c9ra01231a)
Supplement: RA-009-C9RA01231A-s001 [file RA-009-C9RA01231A-s001.pdf]

Electronic Supplementary Material (ESI) for RSC Advances.
This journal is © The Royal Society of Chemistry 2019

Electronic Supplementary Information

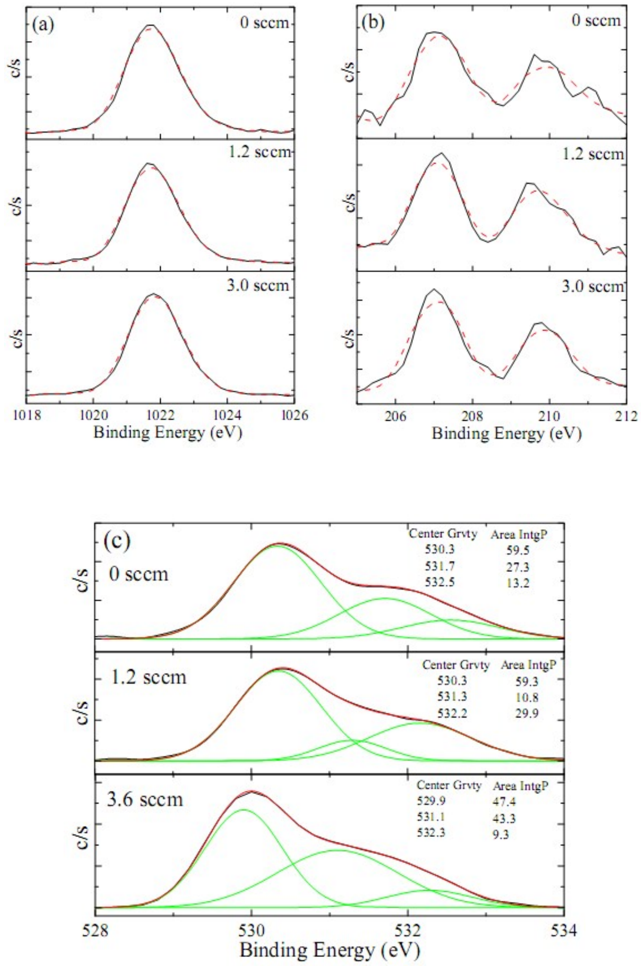

Fig. 4. The typical XPS spectra of (a) Zn 2p, (b) Nb 3d, and (c) O 1s of the NZO films with and without H doping.
